# Prediction of the acceptance of telemedicine among rheumatic patients: a machine learning-powered secondary analysis of German survey data

**DOI:** 10.1007/s00296-023-05518-9

**Published:** 2024-01-11

**Authors:** Felix Muehlensiepen, Pascal Petit, Johannes Knitza, Martin Welcker, Nicolas Vuillerme

**Affiliations:** 1 Univ. Grenoble Alpes, AGEIS, 38000 Grenoble, France; 2 Faculty of Health Sciences Brandenburg, Center for Health Services Research, Brandenburg Medical School Theodor Fontane, Seebad 82/83, 15562 Rüdersdorf bei Berlin, Germany; 3 Institute for Digital Medicine, University Hospital of Giessen and Marburg, Philipps-University Marburg, Marburg, Germany; 4 Medizinisches Versorgungszentrum für Rheumatologie Dr M Welcker GmbH, Planegg, Germany; 5 Institut Universitaire de France, Paris, France; 6 LabCom Telecom4Health, Orange Labs & Univ. Grenoble Alpes, CNRS, Inria, Grenoble INP-UGA, Grenoble, France

**Keywords:** Machine learning, Deep learning, Artificial intelligence, Telemedicine, Digital rheumatology, Primary care, Health Services Research, e-health, Patient, Prediction, Acceptance, Predictors

## Abstract

Telemedicine (TM) has augmented healthcare by enabling remote consultations, diagnosis, treatment, and monitoring of patients, thereby improving healthcare access and patient outcomes. However, successful adoption of TM depends on user acceptance, which is influenced by technical, socioeconomic, and health-related factors. Leveraging machine learning (ML) to accurately predict these adoption factors can greatly contribute to the effective utilization of TM in healthcare. The objective of the study was to compare 12 ML algorithms for predicting willingness to use TM (TM try) among patients with rheumatic and musculoskeletal diseases (RMDs) and identify key contributing features. We conducted a secondary analysis of RMD patient data from a German nationwide cross-sectional survey. Twelve ML algorithms, including logistic regression, random forest, extreme gradient boosting (XGBoost), and neural network (deep learning) were tested on a subset of the dataset, with the inclusion of only RMD patients who answered “yes” or “no” to TM try. Nested cross-validation was used for each model. The best-performing model was selected based on area under the receiver operator characteristic (AUROC). For the best-performing model, a multinomial/multiclass ML approach was undertaken with the consideration of the three following classes: “yes”, “no”, “do not know/not answered”. Both one-vs-one and one-vs-rest strategies were considered. The feature importance was investigated using Shapley additive explanation (SHAP). A total of 438 RMD patients were included, with 26.5% of them willing to try TM, 40.6% not willing, and 32.9% undecided (missing answer or “do not know answer”). This dataset was used to train and test ML models. The mean accuracy of the 12 ML models ranged from 0.69 to 0.83, while the mean AUROC ranged from 0.79 to 0.90. The XGBoost model produced better results compared with the other models, with a sensitivity of 70%, specificity of 91% and positive predictive value of 84%. The most important predictors of TM try were the possibility that TM services were offered by a rheumatologist, prior TM knowledge, age, self-reported health status, Internet access at home and type of RMD diseases. For instance, for the yes vs. no classification, not wishing that TM services were offered by a rheumatologist, self-reporting a bad health status and being aged 60–69 years directed the model toward not wanting to try TM. By contrast, having Internet access at home and wishing that TM services were offered by a rheumatologist directed toward TM try. Our findings have significant implications for primary care, in particular for healthcare professionals aiming to implement TM effectively in their clinical routine. By understanding the key factors influencing patients' acceptance of TM, such as their expressed desire for TM services provided by a rheumatologist, self-reported health status, availability of home Internet access, and age, healthcare professionals can tailor their strategies to maximize the adoption and utilization of TM, ultimately improving healthcare outcomes for RMD patients. Our findings are of high interest for both clinical and medical teaching practice to fit changing health needs caused by the growing number of complex and chronically ill patients.

**Supplementary Information:**

The online version contains supplementary material available at 10.1007/s00296-023-05518-9.

## Background

Telemedicine (TM), which is a natural evolution of healthcare in the digital world, has a crucial role in clinical practice and has the potential to greatly affect public and global health by improving long-term health and increasing access to preventive care. TM, a rapidly evolving field at the intersection of healthcare and technology, has revolutionized the delivery of medical services by enabling remote consultations, diagnosis, treatment and monitoring of patients. It has become a high potential tool in overcoming geographical barriers, improving access to healthcare and enhancing patient outcomes [1]. Despite the potential, the successful implementation of TM stands or falls on the acceptance of the users [2, 3]. This is strongly dependent on the technical, socio-economic and health-related factors [1]. A previous study revealed that patients’ willingness to use TM (TM try) in rheumatology care was associated with several factor such as their perceived health status, age, disease type and access to technical equipment and infrastructure [4].

Yet, it is still unclear which of these factors are good predictors of TM try. For clinicians, reliable predictors of TM try can guide the development of more patient-centered TM services, improving patient engagement and efficiency of care. The identification of such factors, and in particular modifiable predictors, could be paramount and useful for practitioners to help them implement new practices to develop and spread the use of TM among patients with rheumatic and musculoskeletal diseases (RMDs). For researchers, these insights can inspire further studies on the barriers and facilitators to telehealth adoption, contributing to a deeper understanding of the digital transformation of healthcare. Knowledge of these factors will not only help predict patient behavior, but also help shape future telehealth strategies and policies, ultimately benefiting the healthcare system as a whole.

Artificial intelligence (AI) offers valuable opportunities to identify predictors of TM acceptance by analyzing large-scale and diverse datasets and uncovering complex relationships. AI algorithms can identify key predictors, understand their interactions and continuously adapt to new data, aiding in the development of targeted interventions and strategies to increase TM utilization and improve patient outcomes [5]. Scaling up effective TM interventions is essential for long-term population health benefits.

This study aimed to predict TM try among RMD patients and to identify key features contributing to TM try. To that end, we compared 12 machine learning (ML) algorithms using data (secondary analysis) from a German nationwide cross-sectional survey conducted earlier [3].

## Methods

The data utilized in this study has been previously described [3]. In summary, the study presents findings from a secondary analysis of data obtained through a cross-sectional, self-administered paper survey conducted in collaboration with the patient organization German League against Rheumatism (Deutsche Rheuma-Liga, Landesvertretung Brandenburg) and outpatient rheumatologists. This survey was part of an exploratory mixed-methods study spanning over 2 years, aiming to investigate the acceptance, opportunities, and barriers to the implementation of TM [3]. Data collection took place between September 1 and December 30, 2019. The questionnaire’s preliminary version was crafted by a team comprising two healthcare researchers and two rheumatologists. This initial draft was informed by insights gathered from expert interviews [3]. In the subsequent phase, the draft underwent a comprehensive review and modification process at the hands of the German League against Rheumatism (Deutsche Rheuma-Liga, Landesvertretung Brandenburg e.V.). The feedback of the patient representatives was discussed during a teleconference and then incorporated into the questionnaire. To refine the questionnaire further, a pretest involving 30 RMD patients was conducted. This evaluation aimed to assess the questionnaire's clarity, wording and the exhaustiveness of predefined response options. Subsequent to this pretest, minor revisions were applied to enhance its precision and relevance. The final version of the questionnaire (Supplementary Material), spanning five pages, encompassed 24 questions thoughtfully grouped into four essential sections: (1) medical care, (2) technology usage, (3) telemedicine and (4) personal data. Response options were categorized as nominal or ordinal, ensuring a nuanced understanding of participants' experiences. Additionally, the questionnaire featured open-ended queries, encouraging participants to express their thoughts freely. Supplementing the questionnaire was pertinent study information, including a definition of telemedicine illustrated through practical examples: "Telemedicine involves the utilization of information and communication technology in medical treatment to bridge geographical gaps. For instance, this could involve a video consultation with a physician for a visual joint examination or a phone conversation with a doctor to assess the effectiveness of prescribed medication". This contextual information aimed to enrich participants' understanding of the survey's scope and purpose.

The survey's inclusion criteria required participants to meet the following conditions: (1) receiving treatment in rheumatology care; (2) being 18 years or older; and (3) residing in Germany. Sampling was conducted through a non-probability, voluntary approach, which involved collaborating with (1) patient organization German League against Rheumatism's working groups, (2) outpatient rheumatology practices and (3) inpatient rheumatology wards. The questionnaires were provided to representatives from these institutions, who then distributed them to eligible individuals meeting the specified inclusion criteria.

### Data selection/population considered

From the aforementioned German nationwide survey, a dataset of 438 patients in total was available. Each patient answered 24 questions related to socio-demographics and health characteristics. Individuals with missing answer or that answered “do not know” regarding TM try were considered as a distinct category leading to three categories: “yes”, “no” and “not answered/do not know”.

### Statistical analysis

All statistical analyses were performed using R software 4.1.2® (R Core Team, Vienna, Austria) for Windows 10©.

#### Machine learning algorithm selection

To select the best ML algorithm, a subset of the dataset was used, with the inclusion of only RMD patients that answered “yes” or “no” to TM try. For this algorithm selection, 294 patients (67.1%) were considered. A total of 12 ML algorithms were used to identify key features contributing to TM try, namely logistic regression, Lasso regression, ridge regression, support vector machine (SVM) using linear classifier, SVM using polynomial basis kernel, SVM using radial basis kernel, random forest, neural network, AdaBoost, k-nearest neighbors, naive Bayes and extreme gradient boosting (XGBoost).

Downsampling was used to produce a balanced 80%/20% train/test split [6]. The training split was used to generate the learned models, while the testing dataset was used for the validation phase to assess the performance of each model to predict the class labels (answering yes or no to TM try). One-hot encoding was applied to all categorical variables with more than two categories, with missing data considered as a category and the elimination of one category for each factor to avoid multicollinearity [7]. Collinear covariates, with a variance inflation factor > 2.5, were excluded from the analysis [8]. Continuous variables were standardized by removing the mean and scaling them to unit variance before ML [9].

Nested cross-validation was used for each model to limit overfitting [10]. More specifically, each of the models underwent a tenfold cross-validation and the classifier hyperparameters were tuned. A random-search approach for model parameter tuning was used to determine the optimal combination of hyperparameters for maximizing accuracy to generate the best model parameters [7]. To ensure robust results, the cross-validation was performed ten times using a different random number generator seed each time [7].

Evaluation indicators, including area under the receiver operator characteristic (AUROC), precision (positive predictive value), recall (sensitivity), balanced accuracy, F1 measure, kappa, specificity, detection rate, detection prevalence, no information rate, average precision, and prevalence were calculated to compare ML algorithms. The best performing model was selected based on the mean AUROC.

Lasso and ridge regression were performed using the glmnet package version 4.1–6 [11]. The XGBoost algorithm was computed using the xgboost package version 1.6.0.1 [12]. The caret package version 6.0–93 was used to perform all other ML models as well as to calculate the confusion matrix [13]. The pROC package version 1.18.0 [14] was used to calculate the AUROC, while the MLmetrics package version 1.1.1 [15] was used to compute evaluation metrics not provided by the caret package.

#### Identification of TM try predictors

For the best performing ML model, a multinomial/multiclass ML approach was performed with the consideration of the three following classes: “yes”, “no”, “do not know/not answered”. Both one-vs.-one and one-vs.-rest strategies were used. Hence the following binary classifications were performed: no vs. rest, yes vs. rest, not answered/do not know vs. rest, yes vs. no, yes vs. not answered/do not know, and no vs. not answered/do not know. For each classification, the feature importance was investigated using Shapley additive explanation (SHAP) that showed each feature’s impact on the model prediction [16, 17]. This analysis indicates to which extent and in which direction (wanting to use TM *versus* not wanting to use TM) a certain feature influences the ML model.

## Results

### Patients’ characteristics

Among the 438 patients from the nationwide survey, 144 patients (144/438, 32.9%) either did not answer the question regarding TM try (22/438, 5%) or answered with the “do not know” option (122/438, 27.9%). A total of 116 patients (265%) were willing to try TM (i.e. answered yes to the TM try question), while 178 were not (40.6%). Most patients were female (309/438, 70.5%), were on average 59 years old (SD = 14.4) and lived in a provincial town or rural area (254/348, 58.0%). Most of the patients lived up to 5 km from the GP’s office (302/438, 68.9%), with only 6.39% of the patients living more than 15 km from the GP’s office. Most of the patients lived up to 10 km from the rheumatologist’s office (144/438, 32.9%), with 19.9% of the patients living more than 30 km from the rheumatologist’s office. Half of the patients considered they had a bad or very bad health status (205/438, 46.8%), while 41.7% (183/438) considered their health status as moderate. Most patients had a rheumatology treatment (394/438, 90.0%). The most common RMDs were rheumatic arthritis (210/438, 47.9%), arthrosis (104/438, 23.7%), osteoporosis (58/438, 13.2%) and psoriatic arthritis (62/438, 14.2%). Most patients had Internet access at home (357/438, 81.5%) and owned an electronic device (392/438, 89.5%), but did not document their health status (265/438, 60.5%). Most patients had prior TM knowledge (225/438, 51.4%) and electronic contact with physician (336/438, 76.7%).

### Performance of machine learning models

Table 1 presents the evaluation performance metrics for each model.

**Table 1:** Comparison of machine learning algorithms

Evaluation metric	Logistic regression	SVM using linear classifier	SVM using radial basis kernel	SVM using polynomial basis kernel	Naive Bayes	knn	Random forest	Neural network	AdaBoost	Lasso regression	Ridge regression	XGBoost
Accuracy (95% CI)	0.74 (0.61; 0.85)	0.71 (0.57; 0.82)	0.78 (0.65; 0.88)	0.76 (0.63; 0.86)	0.72 (0.59; 0.83)	0.69 (0.56; 0.81)	0.76 (0.63; 0.86)	0.78 (0.65; 0.88)	0.78 (0.65; 0.88)	0.79 (0.67; 0.89)	0.78 (0.65; 0.88)	0.83 (0.71; 0.91)
AUROC (95% CI)	0.79 (0.68; 0.91)	0.79 (0.67; 0.91)	0.86 (0.75; 0.96)	0.86 (0.76; 0.97)	0.85 (0.75; 0.95)	0.86 (0.77; 0.96)	0.88 (0.78; 0.97)	0.83 (0.73; 0.94)	0.87 (0.78; 0.97)	0.85 (0.75; 0.95)	0.85 (0.75; 0.95)	0.90 (0.82; 0.98)
Kappa	0.44	0.35	0.50	0.46	0.37	0.29	0.47	0.50	0.51	0.54	0.51	0.63
No Information Rate	0.60	0.60	0.60	0.60	0.60	0.60	0.60	0.60	0.60	0.60	0.60	0.60
P-Value [Acc > NIR]	0.02	0.07	0.004	0.01	0.04	0.11	0.01	0.004	0.004	0.002	0.004	0.0002
Mcnemar's Test P-Value	0.30	0.15	0.03	0.02	0.006	0.01	0.18	0.03	0.10	0.04	0.10	0.34
Sensitivity/Recall	0.57	0.48	0.52	0.48	0.39	0.35	0.57	0.52	0.57	0.57	0.57	0.70
Specificity	0.86	0.86	0.94	0.94	0.94	0.91	0.89	0.94	0.91	0.94	0.91	0.91
Average precision	0.68	0.68	0.72	0.72	0.71	0.62	0.71	0.70	0.73	0.71	0.71	0.74
Precision/PPV	0.72	0.69	0.86	0.85	0.82	0.73	0.77	0.86	0.81	0.87	0.81	0.84
NPV	0.75	0.71	0.75	0.73	0.70	0.68	0.76	0.75	0.76	0.77	0.76	0.82
*F*1 score	0.63	0.56	0.65	0.61	0.53	0.47	0.65	0.65	0.67	0.68	0.67	0.76
Prevalence	0.40	0.40	0.40	0.40	0.40	0.40	0.40	0.40	0.40	0.40	0.40	0.40
Detection Rate	0.22	0.19	0.21	0.19	0.16	0.14	0.22	0.21	0.22	0.22	0.22	0.28
Detection Prevalence	0.31	0.28	0.24	0.22	0.19	0.19	0.29	0.24	0.28	0.26	0.28	0.33
Balanced Accuracy	0.71	0.67	0.73	0.71	0.67	0.63	0.73	0.73	0.74	0.75	0.74	0.81

*95% CI* 95% confidence interval, *Acc* accuracy, *AUROC* area under the receiver operator characteristic, *knn* k-nearest neighbors, *NIR* no Information Rate, *NPV* negative predictive value, *PPV* positive predictive value

**Fig. 1:**
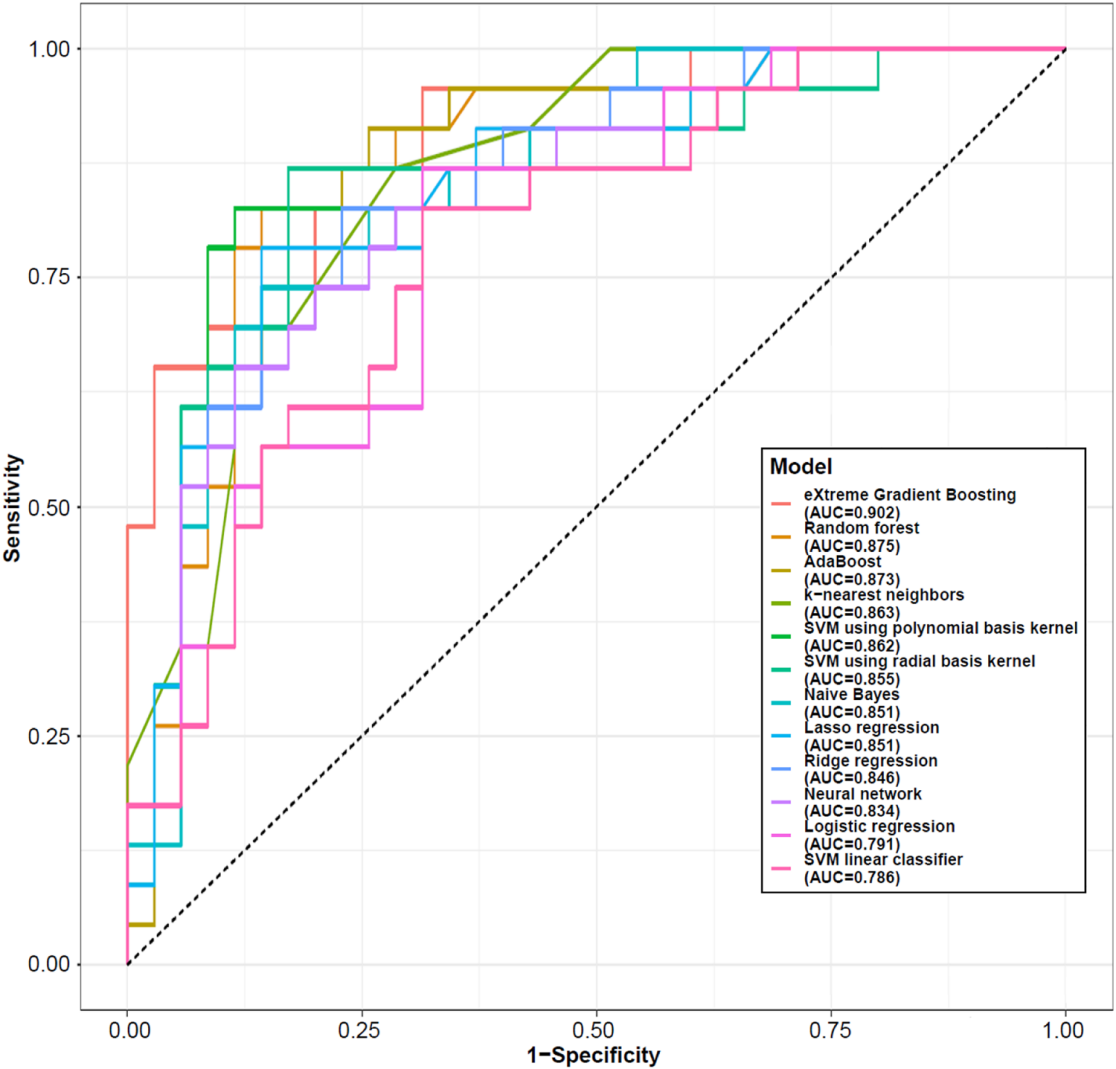
Comparison of machine learning model performance. AUC: area under the receiver operator characteristic

The mean accuracy from the 12 ML models ranged from 0.69 for the k-nearest neighbors model to 0.83 for XGBoost, respectively. The mean AUROC ranged from 0.79 for both logistic regression and SVM linear classifier to 0.90 for XGBoost, respectively (Table 1, Fig. 1). The XGBoost model produced better results compared with the other models, with sensitivity that was 70%, specificity 91% and positive predictive values 84% (Table 1).

The XGBoost model was then used to perform the multiclass classification for all one-vs.-rest and one-vs.-one strategies, yielding an overall AUROC of 0.80 and an accuracy of 0.82. For each binary classification, the AUROC ranged from 0.71 to 0.90 and the accuracy from 0.64 to 0.88 (Table 2).

**Table 2:** results of the multiclass classification

Evaluation metric	Overall	One-vs-Rest			One-vs-One		
		No vs. rest	Yes vs. rest	Not answered vs. rest	Yes vs. no	Yes vs. not answered	No vs. not answered
Accuracy (95% CI)	0.82 (0.75; 0.88)	0.83 (0.76; 0.89)	0.88 (0.81; 0.93)	0.77 (0.69; 0.84)	0.83 (0.71; 0.91)	0.64 (0.59; 0.77)	0.83 (0.71; 0.91)
AUC	0.80 (0.72; 0.88)	0.83 (0.76; 0.90)	0.83 (0.75; 0.91)	0.74 (0.66; 0.82)	0.90 (0.82; 0.98)	0.71 (0.57; 0.85)	0.82 (0.71; 0.92)
Kappa	0.60	0.66	0.68	0.48	0.63	0.28	0.65
No Information Rate	0.41	0.59	0.74	0.67	0.60	0.54	0.53
*P*-value [Acc > NIR]	2.6e-14	1.7e-9	9.3e-5	8.6e-3	2.0e-4	0.11	6.4e-7
Mcnemar's Test *P*-value	0.36	0.83	0.80	1.00	0.34	0.17	0.55
Sensitivity/Recall	0.74	0.82	0.74	0.65	0.70	0.54	0.88
Specificity	0.86	0.84	0.93	0.83	0.91	0.75	0.77
Average precision	0.65	0.51	0.82	0.61	0.74	0.53	0.80
Precision/PPV	0.74	0.79	0.78	0.65	0.84	0.71	0.81
NPV	0.87	0.87	0.91	0.83	0.82	0.58	0.85
*F*1 score	0.74	0.80	0.76	0.65	0.76	0.61	0.85
Prevalence	0.34	0.41	0.26	0.33	0.40	0.54	0.53
Detection Rate	0.26	0.34	0.19	0.21	0.28	0.29	0.47
Detection Prevalence	0.35	0.43	0.24	0.33	0.33	0.40	0.58
Balanced Accuracy	0.80	0.83	0.83	0.74	0.81	0.64	0.83

*95% CI* 95% confidence interval, *Acc* accuracy, *AUROC* area under the receiver operator characteristic, *knn* k-nearest neighbors, *NIR* No Information Rate, *NPV* negative predictive value, *PPV* positive predictive value

### Feature importance

**Fig. 2:**
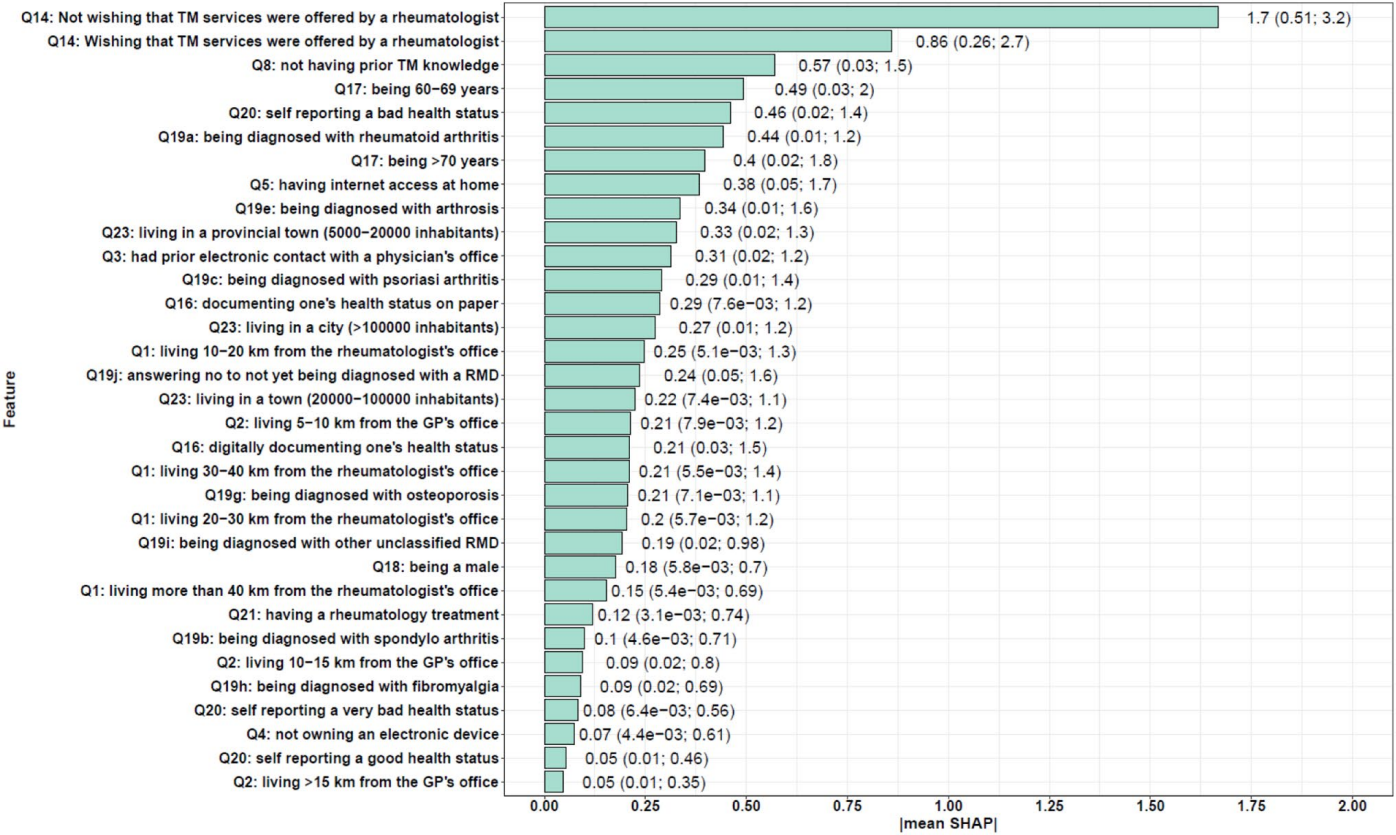
Overall feature importance according to SHAP values

The feature analysis included a total of 28 survey features (answers to 25 questions). Figure 2 lists the most important features using the XGBoost model for the multiclass classification. Most of these features are modifiable. The most important features were the possibility that TM services were offered by a rheumatologist, prior TM knowledge, age, self-reported health status, Internet access at home and type of RMD diseases.

**Fig. 3:**
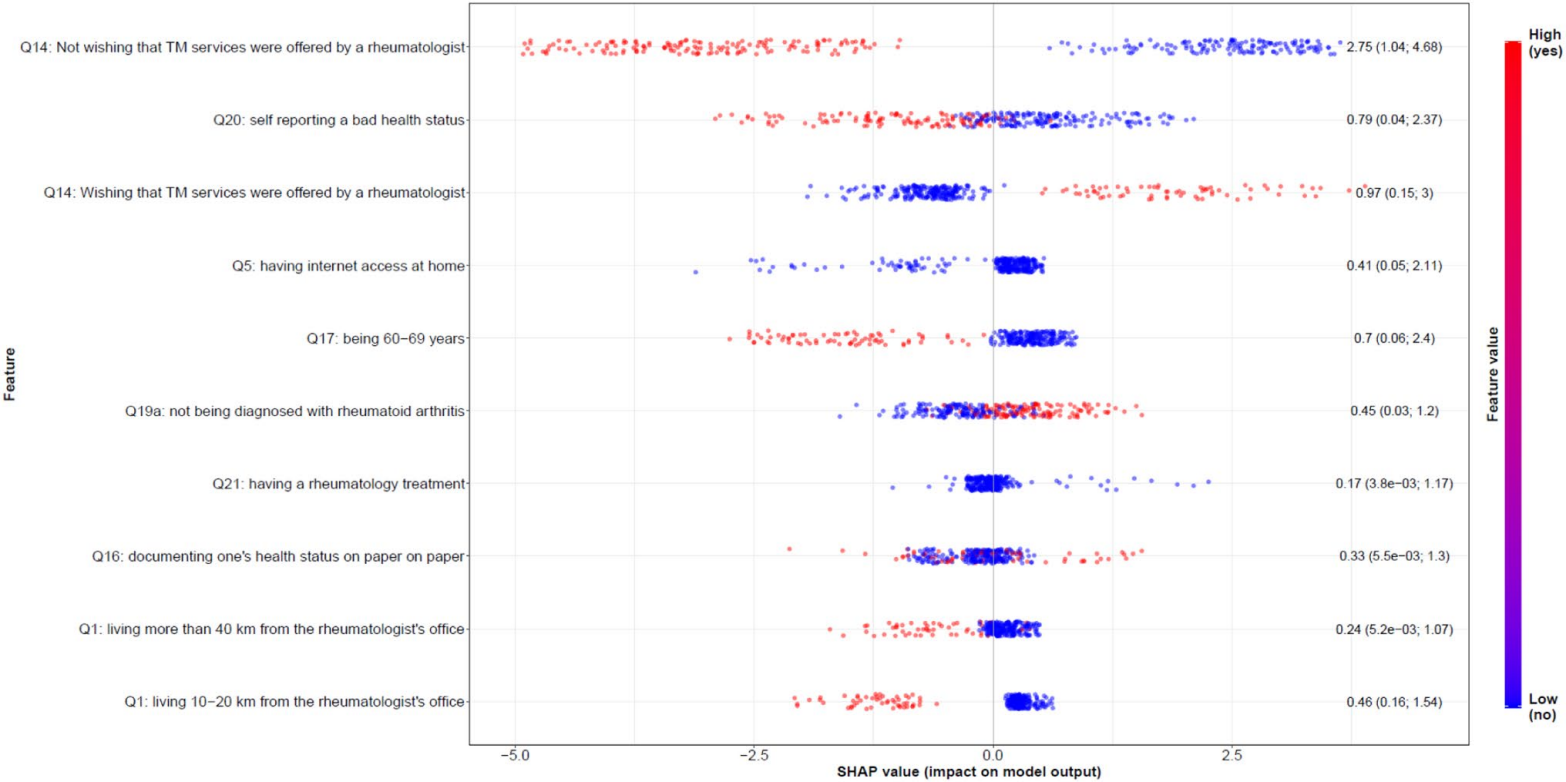
Feature importance (top 10) according to SHAP values for the yes vs. no classification

Regardless of the binary classification considered, results were mostly similar. Figure 3 lists the ten most important features for the yes vs. no classifications. The same figure for all the 28 features is available in the additional file (Fig. S1). The top five predictors of TM try in RMD patients using XGBoost were self-reported wish regarding the possibility that TM services were offered by a rheumatologist, self-reported health status, Internet access at home and age. Most of these features are modifiable. Not wishing that TM services were offered by a rheumatologist was the most important feature by far (mean absolute SHAP value (maSHAP) = 2.75), followed by self-reporting a bad health status (maSHAP = 0.79), being aged 60–69 years (maSHAP = 0.70) and living more than 40 km from the rheumatologist's office (maSHAP = 0.24) or 10–20 km (maSHAP = 0.46). These aforementioned features directed the model toward not wanting to try TM. By contrast, wishing that TM services were offered by a rheumatologist (maSHAP = 0.97), having Internet access at home (maSHAP = 0.41) and not being diagnosed with rheumatoid arthritis (maSHAP = 0.45) were top features directing toward wanting to try TM.

Figures S2–S6 present the list of the most important features for all the other binary classifications. For the yes vs. not answered/do not know classification, the top five predictors of TM try were wishing that TM services were offered by a rheumatologist (positive impact—toward yes), living in a provincial town (positive impact), living in a city (positive impact), living 5–10 km from the GP’s office (negative impact—toward do not know/not answered) and not wishing that TM services were offered by a rheumatologist (negative impact) (Fig. S2).

For the no vs. not answered/do not know classification, the top five predictors of TM try were not wishing that TM services were offered by a rheumatologist (positive impact—toward no to TM try), having Internet access at home (negative impact—toward do not know/not answered), living in a town (positive impact), self-reporting a bad health status (positive impact) and being diagnosed with rheumatoid arthritis (negative impact) (Fig. S3). For the yes vs. rest classification, the top five predictors of TM try were wishing that TM services were offered by a rheumatologist (positive impact—toward yes), not wishing that TM services were offered by a rheumatologist (negative impact—toward no of do not know/not answered), not having prior TM knowledge (negative impact), being age > 60 years (negative impact) and being diagnosed with rheumatoid arthritis (negative impact) (Fig. S4).

For the no vs. rest classification, the top five predictors of TM try were not wishing that TM services were offered by a rheumatologist (positive impact—toward no to TM try), being 60–69 years (positive impact), not having prior TM knowledge (positive impact), having Internet access at home (negative impact—toward yes or do not know/not answered), and being diagnosed with rheumatoid arthritis (positive impact) (Fig. S5).

For the not answered/do not know vs. rest classification, the top five predictors of TM try were not wishing that TM services were offered by a rheumatologist (negative impact—toward no and yes), wishing that TM services were offered by a rheumatologist (negative impact), living in a city (negative impact), self-reporting a bad health status (negative impact), and being diagnosed with arthrosis (negative impact) (Fig. S6).

## Discussion

We have performed a secondary analysis using data from a German nationwide cross-sectional survey among RMD patients in [3] to compare 12 ML algorithms to identify an accurate ML model to predict the patient’s motivation to try TM.

### Principal results

The XGBoost model produced better results compared with the 11 other models tested. XGBoost is a flexible nonlinear tree-based ML algorithm that is commonly used for cohort and survey data in the medical field [6, 18–20]. XGBoost is one of the most widely used methods in data science that has been shown to perform usually better than other ML algorithms in several studies [21, 22].

The XGBoost model analysis revealed that the primary predictors of TM try in RMD patients were identified as self-reported wish for TM services offered by a rheumatologist, self-reported health status, Internet access at home, and age. While age is a non-modifiable factor and self-reported health status changes only during therapy, home Internet access and the desire for the rheumatologist to provide TM can be altered.

### Comparison with prior work

In the field of rheumatology, various ML and deep learning algorithms have been employed for tasks such as clinical profiling, patient classification, diagnosis identification, image analysis and predicting treatment response, primarily for rheumatoid arthritis and systemic lupus erythematosus [10, 19, 20, 23–25]. However, to the best of our knowledge, this study represents the first attempt to utilize ML techniques to predict TM try among RMD patients and identify the key contributing factors based on nationwide survey data. This novel approach extends our understanding of the factors influencing TM adoption in the context of rheumatic diseases.

In other healthcare domains, ML has already been applied to the adoption and acceptance of TM [26–29]. Békes et al. successfully developed a predictive model to understand psychotherapists' acceptance of telepsychotherapy during the COVID-19 pandemic [26]. These authors found that therapists' professional self-doubt and the quality of their working alliance with their online patients appear to be the most relevant factors associated with therapists' acceptance of telepsychotherapy technology. Zobair et al. used a two-staged structural equation modeling and artificial neural network approaches to forecast care seekers' satisfaction with TM [27]. The study results show that enjoyment, which refers to the perception that the use of TM services will be enjoyable or pleasant in its own right, predominantly contributes to patients' satisfaction decision regarding TM usage. This indicates that TM approaches should also provide enjoyment to their users in order to be utilized. This points to the relevance of gamification in the development of eHealth approaches to achieve high adoption rates.

ML approaches also have significant potential in smartphone-based prevention and health promotion to understand user perspectives and tailor targeted interventions to users. For example, ML algorithms have been used by Vera et al. and Etter et al. to identify independent predictors of smoking cessation, smoking reduction and relapse in mHealth users [28, 29]. Another example is the assessment of the popularity and perceived effectiveness of smartphone tools that track and limit smartphone use to promote mental health [30]. Despite the above-mentioned studies, the potential of ML in studying and promoting the use of digital health is far from being fully explored.

### Implications

The self-reported wish for TM services offered by a rheumatologist emerged as the most important predictor of TM try. This underscores the crucial role of healthcare providers in promoting telehealth adoption among their patients. Similar results were reported by Dahlhausen et al. on the adaption of digital therapeutics [31]. Kernder et al. [32] identified a lack of supporting evidence for TM as a main barrier for rheumatologists and poor usability of TM tools as the main barrier from a patient perspective.

These findings highlight significant implications for medical education and training, particularly underscoring the necessity to integrate courses focused on TM, digital health technologies and AI. This integration is crucial to enhance physicians' knowledge and proficiency in utilizing digital technologies. Moreover, it is essential for empowering physicians to effectively propose, elucidate, and engage in discussions about these technologies with their patients. Such an educational advancement is pivotal in aligning medical training with the rapidly evolving digital health landscape. By empowering patients and rheumatologists and ensuring their active involvement in TM initiatives, healthcare systems could tackle workforce shortage while achieving high quality and easy to access rheumatology care [33, 34].

Furthermore, the identified predictors, such as Internet access at home and self-reported health status, offer opportunities for intervention. Initiatives aiming at expanding Internet access and digital literacy can facilitate the adoption of TM services, particularly among patients who currently face barriers related to connectivity, for instance in rural areas. The high relevance of self-perceived health-status on TM acceptance shows that the timing of addressing TM in therapy and appropriateness is crucial. Accordingly, we agree with Rossen et al. that it is important to identify vulnerable sub-populations with particular needs when introducing health technology to offer appropriate medical care and support individuals in taking advantage of technology [35]. Thus, the evaluation of the individuals’ receptiveness to use technology is important to reduce the risk of alienating low-resource individuals before introducing technology into healthcare. Similarly, Kulcsar et al. suggest a triage mechanism to ensure that patients are appropriately paired with the proper type of rheumatology care in the future [36].

ML could play a key role in identifying potential TM users and tailoring TM concepts to the specific needs of patients and healthcare professionals. By addressing these modifiable factors and tailoring interventions accordingly, healthcare providers and policymakers can work collaboratively to increase TM use and enhance patient engagement in the management of rheumatic diseases. This can lead to improve access to specialized care, better disease management, and enhance patient outcomes in the realm of TM.

### Limitations

The relatively small sample size is a limitation that bears an intrinsic risk of over-fitting though we used ten-fold cross-validation as the resampling method to avoid overfitting.

The primary data on which this analysis is based was collected until 30 December 2019, i.e., shortly prior to SARS-CoV-2 outbreak in Germany (27 January 2020). Due to the need to reduced physical contacts and thus minimize the risk of infection, usage of TM initially received a major uptake in global healthcare delivery [37]. Hence, more RMD patients and likely other subgroups will have tried TM by now [38]. A replication of the initial survey is essential to identify whether and how the identified factors have changed. Apart from this, the limitations of the primary data still apply [3]. These are primarily the high potential of self-selection and non-response bias.

## Conclusions

The XGBoost ML model produced the best results to predict patients’ motivation to try TM compared with the 11 other models tested. The model revealed, that the primary predictors of TM try in RMD patients were identified as self-reported wish for TM services offered by a rheumatologist, self-reported health status, Internet access at home and age. Our findings have significant implications for primary care because they emphasize the pivotal role of healthcare professionals in driving the TM implementation and digital transformation in healthcare. By understanding the key factors influencing patients' acceptance of telemedicine, healthcare professionals can tailor their strategies to maximize the adoption and utilization of telemedicine, ultimately improving healthcare outcomes for patients. Our findings are of high interest for both clinical and medical teaching practice to fit changing health needs caused by the growing number of complex and chronically ill patients. By strengthening healthcare professionals and actively engaging them in TM initiatives, health systems could address workforce shortages while providing high-quality and accessible healthcare, which is paramount for long-term population health benefits.

### Supplementary Information

Below is the link to the electronic supplementary material.

Supplementary file1 (PDF 384 KB)

Supplementary file2 (PDF 363 KB)

Supplementary file3 (PDF 386 KB)

Supplementary file4 (PDF 377 KB)

Supplementary file5 (PDF 384 KB)

Supplementary file6 (PDF 567 KB)

## Data Availability

All data relevant to the study are included in the article or uploaded as supplementary material. For further questions regarding the reuse of data, please contact the corresponding author (F.M.).

## Abbreviations

95% CI: 95% Confidence interval
Acc: Accuracy
AUROC: Area under the receiver operator characteristic
e.g.: For example
knn: K-nearest neighbors
maSHAP: Mean absolute SHAP value
ML: Machine learning
NIR: No information rate
NPV: Negative predictive value
PPV: Positive predictive value
RMD: Rheumatic and musculoskeletal disease
SHAP: Shapley additive explanation
SVM: Support vector machine
TM: Telemedicine
TM try: Willingness to use telemedicine
XGBoost: Extreme gradient boosting

## Availability of data and materials

The datasets used and/or analyzed during the current study are available from the corresponding author on reasonable request.
