# Supplementary material for: Prediction of the acceptance of telemedicine among rheumatic patients: a machine learning-powered secondary analysis of German survey data
Source: Rheumatol Int. 2024 Jan 11;44(3):523–34. doi: 10.1007/s00296-023-05518-9 (PMC10866795; doi:10.1007/s00296-023-05518-9)
Supplement: Supplementary file 2 — Supplementary file2 (PDF 363 KB) [file 296_2023_5518_MOESM2_ESM.pdf]

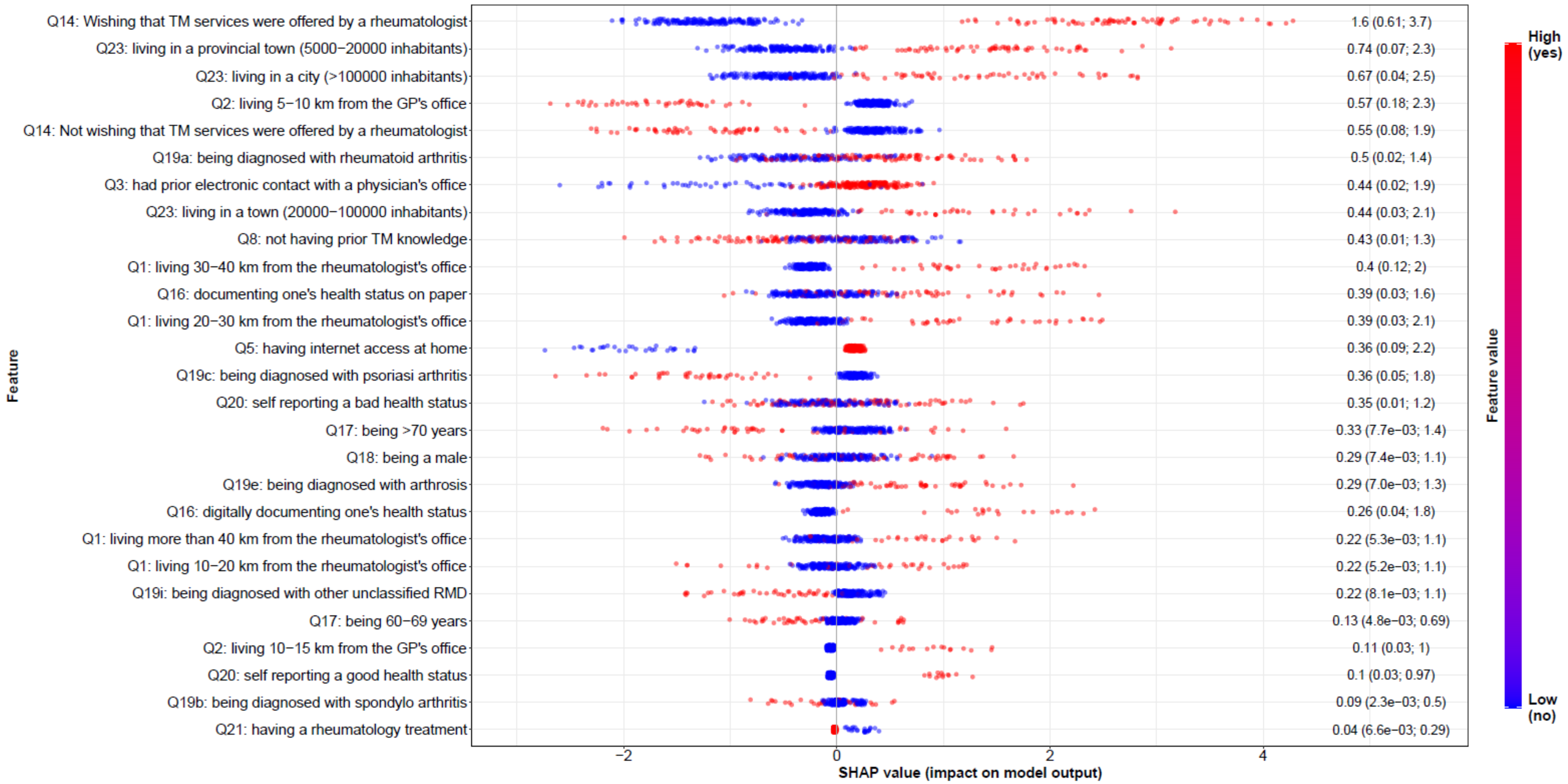

Figure S2: Feature importance according to SHAP values – yes vs. not answered/do not know classification

SHAP value (x-axis) for each patient and feature are represented with a point. Positive SHAP values imply an impact to the model toward wanting to try TM, while negative values impact the model toward not answering/do not know answers regarding TM try. For each feature, the mean and 95% CI of the absolute SHAP values are reported on the right of the graph. High SHAP value (in absolute value) indicate a high impact on the model output. Red colors indicate that a patient answered yes to the considered question (y-axis) while blue colors refer to "no" answers.
