# Supplementary material for: Prediction of the acceptance of telemedicine among rheumatic patients: a machine learning-powered secondary analysis of German survey data
Source: Rheumatol Int. 2024 Jan 11;44(3):523–34. doi: 10.1007/s00296-023-05518-9 (PMC10866795; doi:10.1007/s00296-023-05518-9)
Supplement: Supplementary file 4 — Supplementary file4 (PDF 377 KB) [file 296_2023_5518_MOESM4_ESM.pdf]

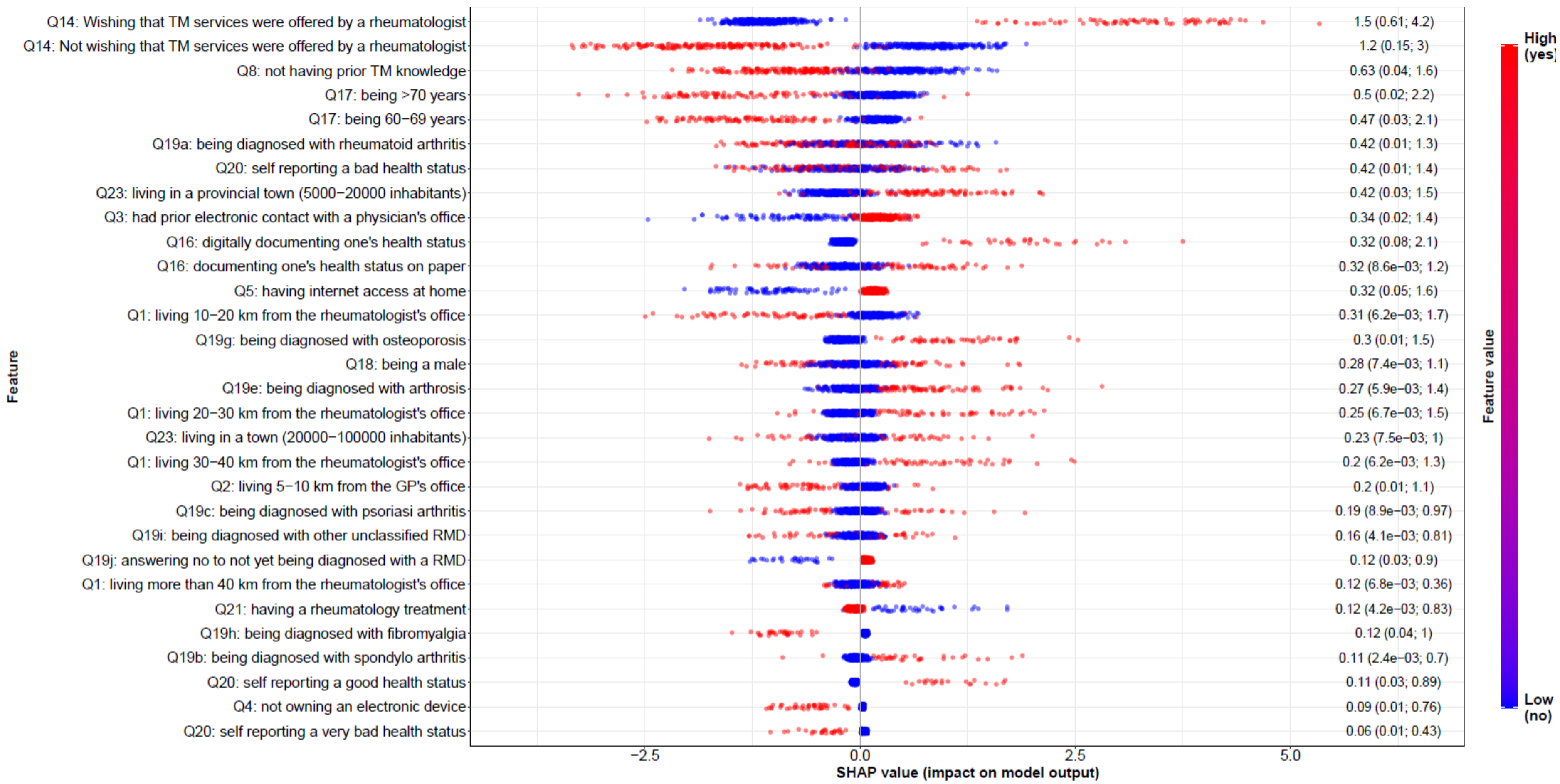

Figure S4: Feature importance according to SHAP values – yes vs. rest classification

SHAP value (x-axis) for each patient and feature are represented with a point. Positive SHAP values imply an impact to the model toward wanting to try TM, while negative values impact the model toward not wanting to try TM or do not know. For each feature, the mean and 95% CI of the absolute SHAP values are reported on the right of the graph. High SHAP value (in absolute value) indicate a high impact on the model output. Red colors indicate that a patient answered yes to the considered question (y-axis) while blue colors refer to “no” answers.
